# Respiratory Syncytial Virus Fusion Protein Promotes TLR-4–Dependent Neutrophil Extracellular Trap Formation by Human Neutrophils

**DOI:** 10.1371/journal.pone.0124082

**Published:** 2015-04-09

**Authors:** Giselle A. Funchal, Natália Jaeger, Rafael S. Czepielewski, Mileni S. Machado, Stéfanie P. Muraro, Renato T. Stein, Cristina B. C. Bonorino, Bárbara N. Porto

**Affiliations:** 1 Clinical and Experimental Immunology Laboratory, Pontifícia Universidade Católica do Rio Grande do Sul (PUCRS), Porto Alegre, RS, Brazil; 2 Cellular and Molecular Immunology Laboratory, Pontifícia Universidade Católica do Rio Grande do Sul (PUCRS), Porto Alegre, RS, Brazil; 3 Infant Center, Pontifícia Universidade Católica do Rio Grande do Sul (PUCRS), Porto Alegre, RS, Brazil; 4 Institute of Biomedical Research, Pontifícia Universidade Católica do Rio Grande do Sul (PUCRS), Porto Alegre, RS, Brazil; University of Georgia, UNITED STATES

## Abstract

Acute viral bronchiolitis by Respiratory Syncytial Virus (RSV) is the most common respiratory illness in children in the first year of life. RSV bronchiolitis generates large numbers of hospitalizations and an important burden to health systems. Neutrophils and their products are present in the airways of RSV-infected patients who developed increased lung disease. Neutrophil Extracellular Traps (NETs) are formed by the release of granular and nuclear contents of neutrophils in the extracellular space in response to different stimuli and recent studies have proposed a role for NETs in viral infections. In this study, we show that RSV particles and RSV Fusion protein were both capable of inducing NET formation by human neutrophils. Moreover, we analyzed the mechanisms involved in RSV Fusion protein-induced NET formation. RSV F protein was able to induce NET release in a concentration-dependent fashion with both neutrophil elastase and myeloperoxidase expressed on DNA fibers and F protein-induced NETs was dismantled by DNase treatment, confirming that their backbone is chromatin. This viral protein caused the release of extracellular DNA dependent on TLR-4 activation, NADPH Oxidase-derived ROS production and ERK and p38 MAPK phosphorylation. Together, these results demonstrate a coordinated signaling pathway activated by F protein that led to NET production. The massive production of NETs in RSV infection could aggravate the inflammatory symptoms of the infection in young children and babies. We propose that targeting the binding of TLR-4 by F protein could potentially lead to novel therapeutic approaches to help control RSV-induced inflammatory consequences and pathology of viral bronchiolitis.

## Introduction

Respiratory Syncytial Virus (RSV)-induced acute bronchiolitis is the most prevalent respiratory disease in children under age 2 years, and its seasonal epidemics are associated with a significant number of hospital admissions, with a huge burden to communities worldwide [1]. Almost 70% of all children are infected with RSV during the first year of life, and by age 3, practically all children will have experienced at least one infection with this virus [2, 3]. RSV is a single stranded RNA virus, whose genome encodes up to 11 proteins [4]. The Fusion (F) protein, present at the virion surface, mediates fusion of the viral envelope with the target cell membrane during virus entry [5]. Only membrane-bound F protein is indispensable for virus replication *in vitro* and *in vivo* [6], and this protein is the primary target for both antiviral drug and vaccine developments [7, 8]. It has been demonstrated that RSV F protein activates pattern recognition receptors TLR-4 and CD14, inducing pro-inflammatory cytokine secretion [9]. In addition, it has been recently shown that RSV F protein directly interacts with the MD-2–TLR-4 complex, thus activating the transcription factor NF-κB [10]. These studies highlight the importance of specific signaling pathways activated by F protein to stimulate inflammation.

One of the characteristic features of RSV infection is the large amounts of neutrophils in the lower airways once infection is established [11]. It is also well recognized that neutrophils and their products are present in the airways of patients and animal models with RSV bronchiolitis [11–13], and also in virus-induced asthma [14, 15]. This body of evidence suggests that neutrophils play an important role in the pathogenesis observed in the airways of affected children [16,17].

Aside from the traditional mechanisms of phagocytosis, generation of reactive oxygen species (ROS), and degranulation, neutrophils can also produce neutrophil extracellular traps (NETs), an important strategy to immobilize and kill pathogens [18]. NETs are formed by decondensed chromatin fibers decorated with antimicrobial proteins, such as neutrophil elastase and myeloperoxidase [18]. NET-inducing stimuli include cell surface components of bacteria, such as LPS, whole bacteria, fungi, protozoan parasites, cytokines, and activated platelets, among others [18–22]. More recently, studies have demonstrated that viruses are also capable of inducing NET formation. *In vitro*, the production of NETs is modulated in neutrophils isolated from cats infected with feline immunodeficiency virus [23]. NETs activated after infection by Human Immunodeficiency Virus (HIV-1) are crucial for the elimination of virus [24]. NET release in the liver vasculature also protects host cells from poxvirus infection [25]. However, an excessive production of NETs contributes to the pathology of respiratory viral infections. NET formation is potently induced in lungs of mice infected with Influenza A virus, in areas of alveolar destruction [26], suggesting a putative role for NETs in lung damage.

We show that RSV virion was able to induce NET formation by human neutrophils and RSV F protein stimulated NET formation dependent on TLR-4 receptor activation. Moreover, F protein-induced NETs were decorated with neutrophil elastase and myeloperoxidase, granule proteins that can damage tissues. F protein potently induced NADPH Oxidase-derived ROS production and this was crucial for NET generation. Also, F protein induced NET production in an ERK and p38 MAPK phosphorylation-dependent manner. Together, these results provide compelling evidence to support a signaling mechanism activated by RSV F protein to induce NET formation. The massive production of NETs in the airways of children infected with RSV may worsen lung pathology and impair lung function.

## Materials and Methods

### Reagents

RSV A2 strain was provided by Dr. Fernando Polack (Vanderbilt University School of Medicine, USA). Human recombinant RSV Fusion protein was purchased from Sino Biological Inc. According to the manufacturer, the glycosylated protein purity is >95% and endotoxin level is <1.0 EU per 1 μg of the protein, as determined by the LAL method. PMA and Protease-free DNase 1 were from Promega. Dextran, LPS O111:B4 from *Escherichia coli*, Diphenyleneiodonium (DPI), N-acetyl-L-cysteine (NAC), and Histopaque-1077 were obtained from Sigma-Aldrich. *ECORI* and *HINDIII* were from Invitrogen. PD98059 and SB203580 were from Cayman Chemical. Polymyxin B and anti-RSV F protein (131-2A) were from Millipore. Blocking anti-TLR-4 (HTA125) and mouse IgG2a isotype control were from eBioscience. The 5-(and-6)-chloromethyl-2’-7’-dichlorodihydrofluorescein diaceate, acetyl ester (CM-H_2_DCFDA) was from Molecular Probes. RPMI 1640 was from Cultilab, and FCS was from Gibco.

### Human neutrophil isolation

Whole blood (20 mL) was collected from healthy volunteer donors (with a mean age of 29 years, from both sexes) with documented verbal consent into heparin-treated tubes. Erythrocytes were removed using Dextran sedimentation followed by two rounds of hypotonic lysis. Neutrophils were isolated from the resulting cell pellet using Histopaque-1077 density centrifugation and then resuspended in RPMI 1640 medium. Neutrophil purity was evaluated by flow cytometry using FACSCanto II (Becton Dickinson), based on morphology and a granulocyte marker expression, resulting in around 97%. Only singlet cells were verified by gating on granulocytes size on the basis of forward scatter (FSC) and side scatter (SSC), followed by CD66b and CD3 expression discrimination. Cell viability was always higher than 99%, as examined by Trypan Blue exclusion assay.

### RSV preparation and neutrophil stimulation

The RSV A2 strain was grown in Hep-2 cells. Virus was purified from cell culture supernatant and the viral titer was determined by infection of Hep-2 cell monolayers followed by a carboxymethylcellulose plaque assay. The virus aliquots were stored in -80°C. Human neutrophils (2 x 10^6^/mL) were stimulated with RSV (10^2^–10^4^ PFU/mL) for 3 h at 37°C under 5% CO_2_ atmosphere. These RSV concentrations were used because higher concentrations were cytotoxic to neutrophils (data not shown). After this period, culture supernatants were collected and NETs were quantified using Quant-iT dsDNA HS kit (Invitrogen), according to manufacturer’s instructions.

### Quantification of NET release

Neutrophils (2 x 10^6^/mL) were stimulated with F protein (0.1–5 μg/mL), LPS (100 ng/mL), PMA (50 nM) or medium alone. After 1 h, 20 U/ml of each restriction enzyme (*ECORI* and *HINDIII*) was added to the cultures, and then kept for 2 h at 37°C, under 5% CO_2_ atmosphere [19]. NETs were quantified in culture supernatants using Quant-iT dsDNA HS kit (Invitrogen), according to manufacturer’s instructions. To evaluate the involvement of TLR-4, NADPHox-derived ROS, and MAPK (ERK and p38) on F protein-induced NET formation, neutrophils were pretreated with selective inhibitors at 37°C under 5% CO_2_, as indicated in figure legends. The Trypan Blue exclusion assay was used to evaluate the viability of cells treated with these inhibitors, and at the end of incubation, the cellular viability was always higher than 97%.

### Immunofluorescence

Neutrophils (2 x 10^5^/300 μL) were incubated with F protein (1 μg/mL), LPS (100 ng/mL), PMA (50 nM) or medium alone for 3 h at 37°C under 5% CO_2_ in 8-chamber culture slides (BD Falcon). After this period, cells were fixed with 4% paraformaldehyde (PFA) and stained with anti-elastase (1:1000; Abcam), followed by anti-rabbit Cy3 antibodies (1:500; Invitrogen) or anti-myeloperoxidase PE antibody (1:1000; BD Biosciences) and Hoechst 33342 (1:2000; Invitrogen). Confocal images were taken in a Zeiss LSM 5 Exciter microscope.

### Assay of intracellular ROS generation

The determination of intracellular ROS generation was based on the oxidation of 0.5 μM 5-(and-6)-chloromethyl-2’,7’-dichlorodihydrofluorescein diacetate, acetyl ester (CM-H_2_DCFDA) to yield an intracellular fluorescent compound. Neutrophils (2 x 10^6^ cells/microtube) were pretreated with NAC (1 mM) or DPI (10 μM) and stimulated with F protein for 60 minutes at 37°C under 5% CO_2_. Afterwards, cells were incubated with CM-H_2_DCFDA for 30 minutes at 37°C under 5% CO_2_. Cytosolic ROS production was measured by flow cytometry using FACSCanto II flow cytometer (Becton Dickinson) with the BD FACSDiva software and analyzed with FlowJo v 7.5.

### Expression of phospho-ERK and phospho-p38

The expression of phospho-ERK 1/2 and phospho-p38 in human neutrophils was measured by flow cytometry using BD Phosflow (BD Biosciences) protocol for human whole blood samples. Neutrophils were stimulated with F protein (1 μg/mL) for 5 minutes. Briefly, cells were fixed in Phosflow Buffer I for 10 minutes at 37°C. After washing, permeabilization was performed with Phosflow Perm Buffer II for 30 minutes on ice. Then, neutrophils were washed twice and stained with APC anti–phospho-ERK 1/2 and Alexa 488 anti–phospho-p38 antibodies for 30 minutes on ice. Also here, data were accessed by flow cytometry using FACSCanto II cytometer (Becton Dickinson) with BD FACSDiva software and analyzed with FlowJo v 7.5.

### Statistical analyses

Data were presented as mean ± SEM. Results were analyzed using GraphPad Prim 5.0 statistical software package. Statistical differences among the experimental groups were evaluated by analysis of variance with Newman-Keuls correction or with Student’s t Test. The level of significance was set at p ≤ 0.05.

### Ethics Statement

This study was reviewed and approved by the Research Ethics Committee of Pontifícia Universidade Católica do Rio Grande do Sul (CEP/PUCRS) under protocol nr. CEP 310.623. CEP/PUCRS approved the use of verbal consent for this study and blood donors provided their verbal informed consent before blood collection. The authors have documented the verbal consent provided by the donors.

## Results

### RSV particles and RSV Fusion protein induce NET formation

**Fig 1:**
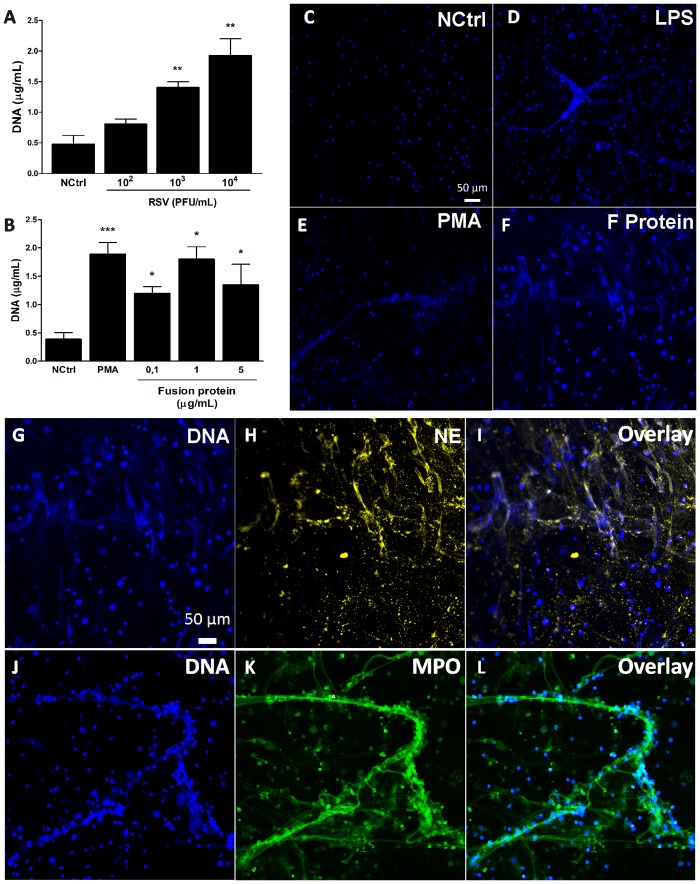
RSV particles and RSV Fusion protein induce NET formation. (A) Human neutrophils (2 x 10^6^/mL) were stimulated with RSV (10^2^–10^4^ PFU/mL) or left unstimulated for 3 h at 37°C with 5% CO_2_. (B) Neutrophils (2 x 10^6^/mL) were stimulated with RSV F protein (0.1–5 μg/mL), PMA (100 nM) or medium alone for 3 h at 37°C with 5% CO_2_. NETs were quantified in culture supernatants using Quant-iT dsDNA HS kit. Data are representative of at least 3 independent experiments performed in triplicates and represent mean ± SEM. *p<0.05; **p<0.01; ***p<0.001 when compared to negative control (NCtrl). (C-F) Neutrophils (2 x 10^5^/300 μL) were stimulated with (C) medium, (D) LPS (100 ng/mL), (E) PMA (100 nM) or (F) F protein (1 μg/mL) for 3 h at 37°C with 5% CO_2_. Cells were then fixed with 4% PFA and stained with Hoechst 33342 (1:2000). Images are representative of at least 4 independent experiments. (G-L) Neutrophils (2 x 10^5^/300 μL) were stimulated with F protein (1 μg/mL) for 3 h at 37°C with 5% CO_2_. Cells were fixed with 4% PFA and stained with: (G-I) Hoechst 33342 (1:2000), anti-elastase (1:1000), followed by anti-rabbit Cy3 (1:500) antibodies; (J-L) Hoechst 33342 (1:2000), anti-myeloperoxidase PE (1:1000) antibody. Overlay of the fluorescence images are shown in the last panels (I,L). Images are representative of 2 independent experiments. Images were taken in a Zeiss LSM 5 Exciter microscope. Scale bars = 50 μm.

It has been previously shown that neutrophils and their products are present in the airways of patients and animals infected with RSV [12–14]. Furthermore, recent studies demonstrated that viruses are able to induce NET formation [24, 25]. Therefore, we sought to investigate whether RSV would be able to induce NET formation in human neutrophils by stimulating neutrophils with increasing concentrations of RSV and quantifying extracellular DNA after 3 h. Indeed, RSV was able to induce NET production in a concentration-dependent manner (Fig 1A). RSV Fusion protein is essential for viral replication [6] and it is known to activate human monocytes, inducing a pro-inflammatory response [9]. We hypothesized that RSV F protein could play a role on NET production. To test that, human neutrophils were stimulated with different concentrations of F protein *in vitro* and after 3 h of incubation extracellular DNA was quantified in culture supernatants. RSV F protein induced NET formation in a dose-dependent manner, with the concentration of 1 μg/mL inducing the strongest response (Fig 1B). In an alternative approach to demonstrate the production of extracellular DNA by F protein, we stimulated neutrophils with medium alone, LPS, PMA or F protein and performed confocal laser scanning microscopy analysis. All stimulants (F protein, PMA and LPS) were able to induce NET formation compared to medium alone (Fig 1C–1F). The expression of antimicrobial proteins on NETs is induced by different stimuli, including bacteria, fungi, and virus [18, 24, 27, 28]. We sought to characterize the composition of NETs induced by RSV F protein, analyzing it by immunostaining. F protein induced the formation of NETs containing the proteins from azurophilic granules, neutrophil elastase (NE) (Fig 1G–1I) and myeloperoxidase (MPO) (Fig 1J–1L), which co-localized with extracellular DNA.

### Effect of different treatments on F protein-induced NETs generation

**Fig 2:**
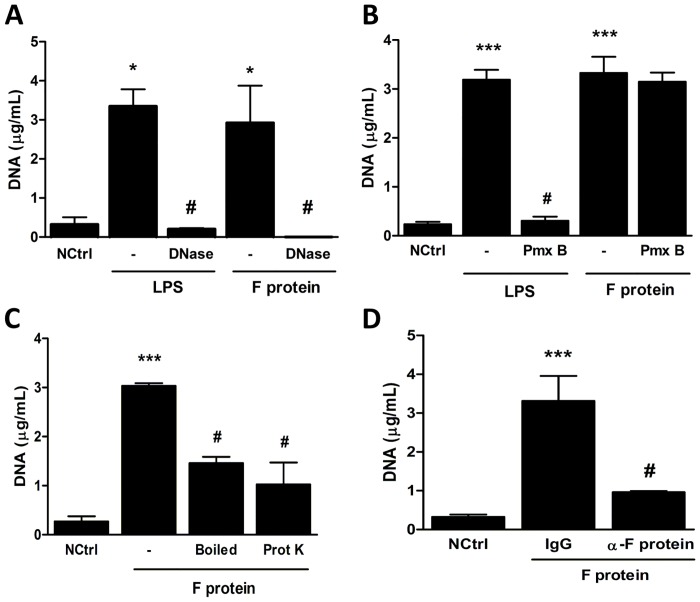
Effect of different treatments on F protein-induced NETs generation. Human neutrophils (2 x 10^6^/mL) were stimulated with: (A) F protein (1 μg/mL) or LPS (100 ng/mL) in the presence or absence of DNase-1 (100U/mL); (B) F protein (1 μg/mL) or LPS (100 ng/mL) in the presence or absence of polymyxin B (Pmx B, 1 μg/mL); (C) F protein (1 μg/mL), boiled F protein (1 μg/mL, 10 min at 100°C) or F protein (1 μg/mL) treated with proteinase K (1 mg/mL for 90 min) for 3 h at 37°C with 5% CO_2_. (D) F protein solution was treated with monoclonal anti-F protein (10 μg/mL) or isotype-matched (10 μg/mL) antibody and neutrophils (2 x 10^6^/mL) were stimulated with these preparations for 3 h at 37°C with 5% CO_2_. NETs were quantified in culture supernatants using Quant-iT dsDNA HS kit. Data are representative of at least 2 independent experiments performed in triplicates and represent mean ± SEM. *p<0.05; ***p<0.001 when compared to negative control (NCtrl); #p<0.05 when compared to LPS- or F protein-treated cells.

To ensure that the structures visualized and quantified were in fact NETs, we stimulated neutrophils with F protein or LPS, as a control, and treated the cells with protease-free DNase. DNase treatment was able to dismantle NETs induced by both LPS and F protein (Fig 2A), indicating that those structures were made of DNA, and consequently NETs. A major concern when characterizing any putative activator of TLR is the possible presence of microbial-derived contaminants. LPS is the prototype TLR-4 agonist and it is among the most potent proinflammatory stimuli both *in vivo* and *in vitro*. To test whether the effect of F protein could be due to LPS contamination, we stimulated neutrophils with F protein or LPS in the presence or absence of polymyxin B and quantified extracellular DNA in culture supernatants. As expected, LPS-induced NET release was inhibited by polymyxin B, which has been previously shown to bind and neutralize LPS [29]. In contrast, F protein was able to induce NET formation in the presence of polymyxin B (Fig 2B), indicating that the effect of F protein is not attributable to LPS contamination. Next, we treated F protein with proteinase K for 90 minutes, to digest the protein structure, or boiled F protein for 10 minutes at 100°C, to further exclude the possibility that the effect could be due to other heat-resistant contaminant. Both treatments profoundly inhibited F protein-induced NET formation (Fig 2C), confirming that only integral F protein is capable of inducing NET production. Finally, we treated the F protein solution with either a neutralizing antibody directed to RSV F protein or with an isotype-matched antibody and stimulated neutrophils with these preparations. The neutralized F protein was not able to induce NET release compared to the protein treated with the control antibody (Fig 2D), confirming its role on NET production.

### F protein-induced NET formation is dependent on TLR-4 activation

**Fig 3:**
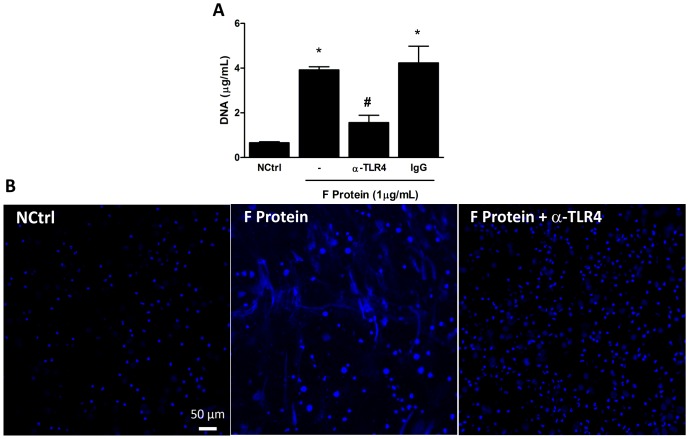
F protein-induced NET formation is dependent on TLR-4 activation. (A) Human neutrophils (2 x 10^6^/mL) were pretreated with monoclonal anti-TLR4 (10 μg/mL) or isotype-matched (10 μg/mL) antibody for 1 h and stimulated with F protein (1 μg/mL) or medium for 3 h at 37°C with 5% CO_2_. NETs were quantified in culture supernatants using Quant-iT dsDNA HS kit. Data are representative of at least 3 separate experiments performed in triplicates and represent mean ± SEM. *p<0.001 when compared to negative control (NCtrl); #p<0.05 when compared to F protein-treated cells. (B) Neutrophils (2 x 10^5^/300 μL) were pretreated with anti-TLR4 (10 μg/mL) for 1 h at 37°C with 5% CO_2_ and stimulated with F protein (1 μg/mL) or medium for 3 h at 37°C with 5% CO_2_. Cells were fixed with 4% PFA and stained with Hoechst 33342 (1:2000). Confocal images were taken in a Zeiss LSM 5 Exciter microscope. Image is representative of 2 independent experiments. Scale bars = 50 μm.

RSV F protein activates the pattern recognition receptors TLR-4–CD14–MD-2 to induce the activation of the transcription factor NF-κB and proinflammatory cytokine secretion [9, 10]. We hypothesized that F protein could activate TLR-4 to induce NET production. A blocking antibody against TLR-4 was used to define the involvement of this receptor on F protein-induced NET formation. Pretreatment of neutrophils with anti-TLR4 significantly inhibited the effect of F protein on NET release (Fig 3A). As an alternative approach to show the role of TLR-4 on NET formation by F protein, we visualized DNA fibers after pretreatment of cells with anti-TLR4. The release of DNA induced by F protein after pretreatment with the antibody is completely blocked (Fig 3B). These results indicate that RSV F protein induces NET formation via a TLR-4 activation pathway.

### Essential role for NADPH Oxidase-derived ROS on F protein-induced NET generation

**Fig 4:**
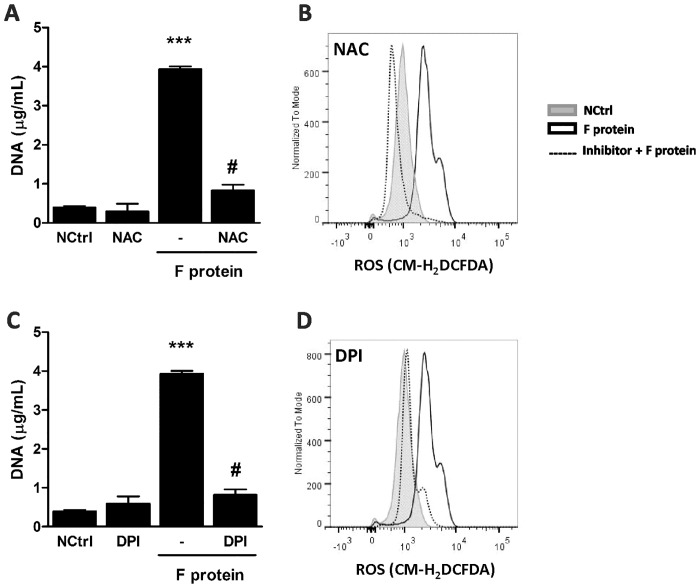
Essential role for NADPH Oxidase-derived ROS on F protein-induced NET generation. (A,C) Neutrophils (2 x 10^6^/mL) were pretreated with NAC (1 mM) or DPI (10 μM) for 1 h and stimulated with F protein (1 μg/mL) for 3 h at 37°C with 5% CO_2_. NETs were quantified in culture supernatants using Quant-iT dsDNA HS kit. Data are representative of 3 separate experiments performed in triplicates and represent mean ± SEM. ***p<0.001 when compared to negative control (NCtrl); #p<0.001 when compared to F protein-treated cells. (B,D) Neutrophils (2 x 10^6^/microtube) were pretreated with NAC (1 mM) or DPI (10 μM) for 1 h, stimulated with F protein (1 μg/mL) for 1 h at 37°C with 5% CO_2_ and incubated with 0.5 μM CM-H_2_DCFDA for 30 min. ROS generation was analyzed by flow cytometry using FACSCanto II flow cytometer. Neutrophils gate was based on FSC x SSC distribution. Data are representative of 2 independent experiments performed in triplicates with similar results.

NET release induced by various agents has been previously shown to depend on ROS generation [27, 30, 31]. To characterize the involvement of ROS on F protein-induced NET production, neutrophils were treated with inhibitors of ROS generation. Treatment with NAC blocked NET formation induced by F protein (Fig 4A) and abrogated F protein-induced ROS generation (Fig 4B). Similarly, treatment with DPI, a NADPH Oxidase inhibitor, significantly inhibited F protein-stimulated NET production (Fig 4C) and abolished ROS generation induced by F protein (Fig 4D). Together, these results indicate that F protein stimulates NET production dependent on NADPH Oxidase-derived ROS generation.

### F protein activates ERK and p38 MAPK to induce NET formation

**Fig 5:**
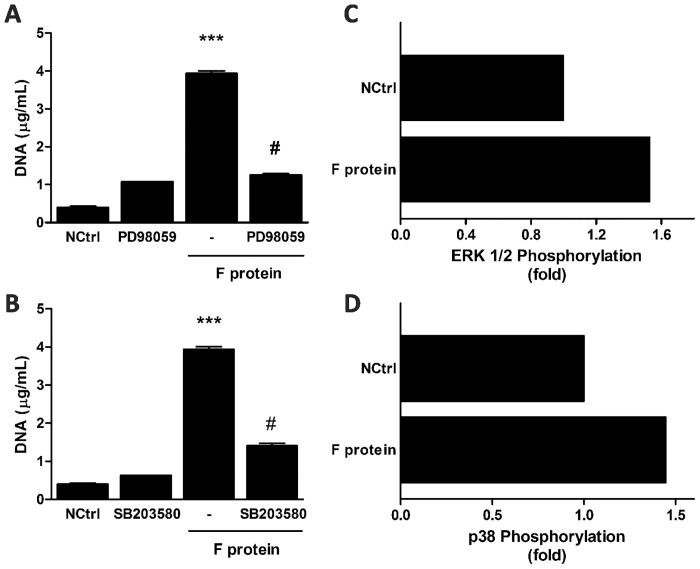
F protein activates ERK and p38 MAPK to induce NET formation. (A,B) Neutrophils (2 x 10^6^/mL) were pretreated with PD98059 (30 μM) or SB203580 (10 μM) for 1 h and stimulated with F protein (1 μg/mL) for 3 h at 37°C with 5% CO_2_. NETs were quantified in culture supernatants using Quant-iT dsDNA HS kit. Data are representative of 3 separate experiments performed in triplicates and represent mean ± SEM. ***p<0.001 when compared to negative control (NCtrl); #p<0.001 when compared to F protein-treated cells. (C,D) Neutrophils (1 x 10^6^/mL) were stimulated with F protein (1 μg/mL) for 5 min at 37°C with 5% CO_2_ and stained for phosphorylated proteins (ERK 1/2 and p38 MAPK), according to Materials and Methods. Proteins phosphorylation was analyzed by flow cytometry using FACSCanto II flow cytometer. Neutrophils gate was based on FSC x SSC distribution. Phosphorylation of protein pathways are presented as fold increase relative to unstimulated neutrophils (NCtrl). Data are representative of 2 separate experiments with similar results.

Recent studies have shown that ERK and p38 MAPK are indispensable for NET production [32,33]. To investigate the role of these MAPK on F protein-induced NET formation, we treated neutrophils with selective inhibitors of ERK and p38 MAPK. Pretreating neutrophils with PD98059 and SB203580, ERK and p38 inhibitors respectively, profoundly decreased DNA release induced by F protein (Fig 5A and 5B), pointing to a critical role for these MAPK on F protein-induced NET formation. We also evaluated whether treatment of neutrophils with F protein would activate these signaling pathways, analyzing phosphorylation of ERK 1/2 and p38. F protein rapidly activated phosphorylation of these signaling pathways (Fig 5C and 5D) leading to NET release, thus supporting the results obtained with the inhibitors.

## Discussion

Neutrophils are key players in microbial containment due to their phagocytic properties, being able to deliver antimicrobial molecules in the phagolysosome and release neutrophil extracellular traps that entrap and kill a multitude of microorganisms [18, 34]. NETs are formed by a variety of stimuli, including bacteria, fungi, parasites, cytokines and endogenous proteins [18, 35–37]. Recent studies proposed a role for NETs in the control of viral infections [24, 25]. Neutrophil-derived NETs were able to capture HIV-1 particles and this effect was dependent on TLR-7 and TLR-8 activation [24]. Systemic injection of viral TLR ligands or poxvirus infection led to accumulation of neutrophils in liver sinusoids that formed aggregates with platelets and released NETs into the vessels [25]. These studies point out to a beneficial role for NETs in controlling and neutralizing viral infection. However, the excessive formation of NETs could be pathogenic to the host, mainly in respiratory viral infections, because NETs could expand more easily in the pulmonary alveoli, causing lung injury. It has been recently shown that Influenza A virus induced the formation of NETs, entangled with alveoli in areas of tissue injury, suggesting a potential link with lung damage [26].

In this study we were able to demonstrate that RSV particle and one of its membrane-expressed glycoproteins potently induced NET formation. RSV F protein caused the release of NETs coated with granular proteins NE and MPO. These proteins have been shown to be important for NET formation [38, 39] and to possess microbicidal activities [21, 24, 40]. MPO present in NETs provides the bactericidal activity against *S*. *aureus* [40] and promotes the elimination of HIV-1 [24]. NE expressed in NETs induced by the pathogenic mold *A*. *fumigatus* helps to inhibit its growth [21]. However, the antimicrobial proteins released with NETs are directly toxic to tissues and the massive production of NETs may damage host tissues [41], as is the case for elastase, which cleaves host proteins at the site of inflammation or infection [42]. Neutrophils actively producing NETs in the lung tissue disturb microcirculation and elicit pulmonary dysfunction [43]. Moreover, NETs directly induce epithelial and endothelial cell death [44]. NE and MPO expressed on DNA fibers stimulated by F protein could exacerbate lung pathology induced by RSV infection, through the destruction of connective tissue, degradation of endothelial cell matrix heparan sulfate proteoglycan, resulting in post infection tissue injury [43].

The fibrous structure of NETs is essential for providing high local concentrations of antimicrobial proteins [45], but it can also be detrimental for host tissues, since it can impair lung function [46]. Furthermore, the characterization of NETs structure is a great concern when studying these DNA lattices and their function. With two different approaches, the quantification of extracellular DNA and fluorescence microscopy, we demonstrated that RSV F protein-induced NETs were dismantled by DNase treatment, confirming that their structural backbone is chromatin.

Together with G protein, F protein comprises the major glycoprotein on RSV surface and these proteins are the main targets of neutralizing antibodies against RSV. F protein mediates the fusion of virus with the target cell and it is essential for viral replication both *in vivo* and *in vitro* [6], being considered the primary target for vaccine and antiviral drug development. Monoclonal antibodies to F protein passively protect against RSV challenge in an animal model and reduce the severity of infection in premature and newborn babies [47, 48]. A major feature of RSV infection is the large numbers of neutrophils recruited to the airways of patients and animals [11–13, 49]. This phenomenon is more profound than in any other respiratory viral infection in childhood, in which mostly alveolar macrophages and T cells prevail.

Although neutrophils are essential effector cells of the innate immune system and have a crucial role in the clearance of microorganisms [50], it has been suggested that neutrophils may contribute to the pathology observed in the airways of patients and animals infected with RSV [51]. Moreover, it has been shown that RSV is able to activate neutrophils, inducing degranulation and IL-8 secretion [52] and also inhibit neutrophil spontaneous apoptosis [53]. It is plausible to reason that these effects could be mediated by F protein binding to TLR-4, once it has been demonstrated that F protein binds to TLR-4/CD14 and physically interacts with MD-2, an essential accessory molecule for TLR-4 activation [9, 10]. F protein induced NET formation in a TLR-4-dependent manner, since the treatment of neutrophils with a blocking antibody against TLR-4 profoundly inhibited extracellular DNA production. Our findings are in agreement with studies showing the activation of TLR-4 by different stimuli to induce NET generation [18, 20, 35]. Importantly, the activation of TLR-4 by F protein was not attributable to LPS contamination, since the treatment with polymyxin B did not inhibit NET formation induced by the protein, but did inhibit the effect of LPS on NET generation. Furthermore, the native conformation of F protein was required in order to stimulate NET formation, once the boiled or proteinase K-digested protein lost this effect, as well as the neutralized protein by a monoclonal antibody directed against RSV F protein.

Stimulation of TLR-4 initiates a signal transduction cascade that induces the assembly of NADPH Oxidase complex. Several studies indicate that ROS are required for NET formation [27, 30, 31]. Then, we sought to investigate whether F protein would be able to stimulate ROS production in neutrophils and whether this induction would be necessary for NET generation. Treatment with the ROS scavenger NAC abolished F protein-induced ROS and extracellular DNA production. Also, the oxidase inhibitor DPI, at the typical concentration needed to block the respiratory burst, completely blocked ROS production and NET formation induced by F protein. Thus, F protein-induced NET release is mediated by ROS generation. How ROS production contributes to DNA release is a question still open for debate. One possibility is that they promote the morphological changes seen in neutrophils secreting NETs [38]. In addition, it has been suggested that ROS can act as second messengers [54]. The requirement of ROS for NET generation induced by RSV F protein indicate that ROS act as second messengers for this stimulus, likely promoting downstream events that culminate in DNA release.

Recent evidence shows that NET formation needs additional signaling, with the involvement of ERK and p38 MAPK. Furthermore, activation of these MAP kinases is downstream of NADPH Oxidase-derived ROS production [32, 33]. We hypothesized that F protein would activate ERK and p38 MAPK to stimulate extracellular DNA release. Treatment of neutrophils with selective inhibitors of ERK and p38 MAPK almost abolished NET induction by F protein. Importantly, F protein was able to activate the phosphorylation of these MAP kinases. Taken together, these results indicate that RSV F protein-induced NET formation is mediated by the phosphorylation of p38 MAPK and ERK.

**Fig 6:**
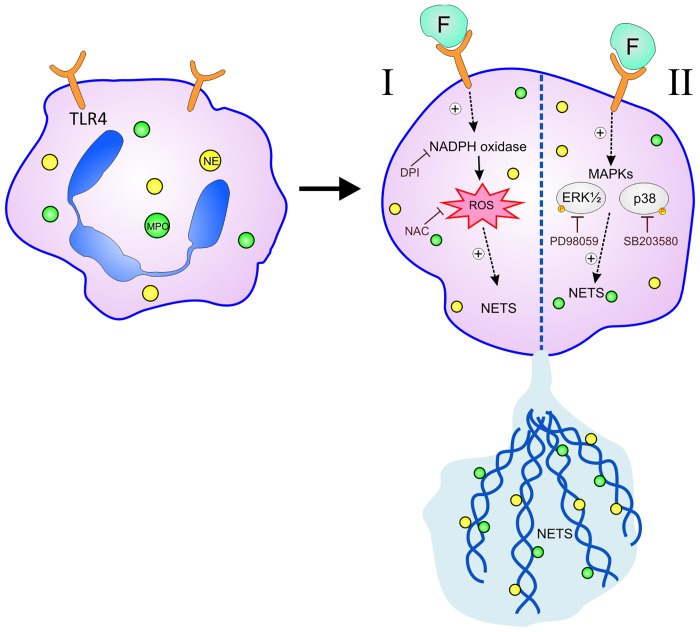
Mechanisms involved in RSV Fusion protein-induced NET formation in human neutrophils. (I) RSV F protein binds to and activates TLR-4, expressed by neutrophils, stimulating ROS production via NADPH Oxidase, which is essential for NET formation. (II) F protein is also able to activate ERK and p38 MAPK to induce NET release. RSV F protein stimulates the production of NETs decorated with the granular proteins NE and MPO.

In conclusion, our study demonstrates that RSV particle stimulates NET formation by human neutrophils and RSV F protein is able to induce NET release through specific signaling pathways. This induction occurs through activation of TLR-4 and it is dependent on NADPH Oxidase-derived ROS generation, and on ERK and p38 MAPK phosphorylation (Fig 6). Neutrophils play an important role in the immunopathology during RSV infection and are continuously recruited from the bone marrow and blood stream to the lungs. The binding of RSV F protein to TLR-4 on neutrophils could induce the massive production of NETs, which can fill the lungs and impair lung function and consequently aggravate the inflammatory symptoms of the infection in young children and babies. We propose that targeting the binding of TLR-4 by F protein or even the associated use of DNase could potentially lead to novel therapeutic approaches to help control RSV-induced inflammatory consequences and pathology of viral bronchiolitis, which has a major disease burden among infants, worldwide.
